# Correlation of 2 Hour, 4 Hour, 8 Hour and 12 Hour Urine Protein with 24 Hour Urinary Protein in Preeclampsia 

**Published:** 2014-09

**Authors:** Savita Rani Singhal, Veena Ghalaut, Shashi Lata, Himanshu Madaan, Veenu Kadian, Ashuma Sachdeva

**Affiliations:** 1 Department of Obstetrics and Gynecology, Pt B D Sharma Post Graduate Institute of Medical Sciences, Rohtak, Haryana, India; 2 Department of Biochemistry, Pt B D Sharma Post Graduate Institute of Medical Sciences, Rohtak, Haryana, India

**Keywords:** Pre Eclampsia, Urinary Protein, Timed Collection

## Abstract

**Objective:** To find shortest and reliable time period of urine collection for determination of proteinuria.

**Materials and methods:** It is a prospective study carried out on 125 pregnant women with preeclampsia after 20 weeks of gestation having urine albumin >1 using dipstick test. Urine was collected in five different time intervals in colors labeled containers with the assistance of nursing staff; the total collection time was 24 hours. Total urine protein of two-hour, four-hour, eight-hour, 12-hour and 24-hour urine was measured and compared with 24-hour collection. Data was analyzed using the Pearson correlation coefficient.

**Results:** There was significant correlation (p value < 0.01) in two, four, eight and 12-hour urine protein with 24-urine protein, with correlation coefficient of 0.97, 0.97, 0.96 and 0.97, respectively. When a cut off value of 25 mg, 50 mg. 100 mg, and 150 mg for urine protein were used for 2-hour, 4-hours, 8-hour and 12-hour urine collection, a sensitivity of 92.45%, 95.28%, 91.51%, and 96.23% and a specificity of 68.42%, 94.74%, 84.21% and 84.21% were obtained, respectively.

**Conclusion:** Two-hour urine proteins can be used for assessment of proteinuria in preeclampsia instead of gold standard 24-hour urine collection for early diagnosis and better patient compliance.

## Introduction

Preeclampsia is a commonly encountered complication of pregnancy accounting for 4-8% of all pregnancies (1). It is defined as systolic blood pressure (BP) level of 140mmHg or higher and diastolic BP of 90mmHg or higher, occurring after 20 weeks of gestation with proteinuria to the extent of >0.3gm protein in 24-hour urine specimen (2). It is a leading cause of maternal and perinatal morbidity and mortality worldwide (3).

Although proteinuria is central to the diagnosis and assessment of severity of preeclampsia, the methods of recording its presence or extent are poorly described or standardized. The dipstick estimation of spot urine samples is the most commonly used and recorded method (4). However, this method is neither sensitive nor specific. Small amounts of protein may be found in alkaline urine, urinary tract infection or contamination by vaginal discharge.

Another method is protein/creatinine ratio of single urine sample, but it also shows a poor correlation (5). The gold standard for urinary protein measurement, till date, is the measurement of 24-hour urine protein (2, 6). The rationale behind 24-hour collection is that in preeclamptic patients, fluctuation of protein loss in urine varies significantly over a 24-hour period and collection of less than this duration may not accurately reflect the actual amount of daily protein loss. But even with this method, there is a major drawback, as it requires too much time for collection and is cumbersome for the patient leading to poor patient compliance (7). Moreover, estimation of 24-hour urine protein may be incorrect due to improper collection, improper mixing or spillage, and it also delays the diagnosis, and hence the treatment.

Efforts are going on to find the shortest and most reliable time period for urine collection, and few studies have been carried out in this regard (8). There is no study available showing the correlation of 2-hour, 4-hour, 8-hour, 12-hour and 24-hour urine protein collectively, so the present study was carried out to correlate the urinary protein levels in 2-, 4-, 8-, and 12-hour urine samples with 24-hour urine sample in preeclampsia.

## Materials and methods

This prospective study was carried in the department of obstetrics and gynecology at a tertiary care hospital, Pt B D Sharma Post Graduate Institute of Medical Sciences, Rohtak, India, in 125 pregnant women admitted with features of preeclampsia after ethical clearance. All the women over 20 weeks gestation with preeclampsia, with blood pressure recording of ≥ 140/90mmHg, and with +1 or more proteinuria by dipstick method were included in the study. Women who had urinary tract infection, preexisting renal disease, chronic hypertension and who delivered before 24 hours were excluded. 

After the informed consent, the women were subjected to a detailed history and clinical examination. The investigations for preeclampsia like renal function test, liver function test and coagulation profile were carried out. Along with these investigations, the quantization of urine was also done.

***Sample collection***

Urine samples were collected in five different time intervals in colors labeled wide mouthed containers. All the patients were assisted by the nursing staff for the collection procedure. The collection of urine started in the morning after discarding the first voided sample and the time of voiding was noted down. The five containers, first to fifth had the urine collected for the first 2-hour, the next 2 hours, the next 4 hours, the next 4 hours and the next 12 hours, respectively, and the total collection period was 24 hours.

The volume of the first two-hour urine was measured, and six ml of this urine was taken as sample-1. The urine from the second container was then added to first container and total volume of four-hour collection was noted down and sample-2 was taken. This four-hour collection was then mixed with the next four-hour collection of container three to get eight-hour collection, and then, sample-3 was taken. The next four-hour collection from container four was then added to this eight-hour collection to get twelve-hour collection, and sample-4 was taken. This twelve-hour collection was added to the next twelve-hour collection (Container Five) to get the twenty four-hour collection, and after noting down the total volume, sample-5 was taken. Each sample taken was six ml. These samples were estimated for urine protein by nephelometry method. The patients were managed as per the existing protocol of the hospital and were followed till delivery. The data was analyzed using the Pearson correlation coefficient.

## Results

Results are shown in the table 1-2. Most of the patients (86.40%) were in the age group of 21-30 years, and the mean age was 24.58 ± 3.9 years. The mean gestational age was 34.88 ± 3.02 weeks, while 97.6% of the subjects had gestational age ≥ 28 weeks. The average systolic and diastolic blood pressure was 151.2 ± 9.6 and 99.02 ± 7.82 mmHg respectively. The systolic blood pressure was >160mmHg in 12.0% of the patients, and diastolic blood pressure was >110mmHg in 8.8% of the patients. The level of proteinuria was either 1+ or 2+ in 92% of cases, and only, 8% of the cases had proteinuria of 3+. This is because, in most of the cases of severe proteinuria, the pregnancy was terminated within 24 hours, and these patients were not included in the study. As far as severity is concerned, two patients developed eclampsia and renal and liver functions were deranged in six patients. Five women had abruption placenta and seven had postpartum hemorrhage. A cutoff value of 25mg, 50mg, 100mg and 150mg for urine protein were used for 2- hour, 4- hour, 8- hour and 12- hour urine collection. ROC curves were obtained and area under curve was 0.95, 0.88, 0.76 and 0.65 for 2, 4, 8 and 12 hour urine protein respectively.

**Table 1:** Distribution of patients according to various parameters

**Parameters**		**n (%)**	**Mean ± SD**
Booking Status	Booked	60 (48%)	-----
	Unbooked	65 (52%)	
Residence	Rural	85 (68%)	----
	Urban	40 (32%)	
Age (years)	<20	7 (5.60%)	24.58 ± 3.90
	21-30	108 (86.40%)	
	>30	10 (08%)	
Gestational Age (Weeks)	<28	3 (2.40%)	34.88 ± 3.02
	29-36	75 (60%)	
	>37	47 (37.60%)	
Proteinuria	1+	84 (67.20%)	-----
	2+	31(24.80%)	
	>3+	10 (8%	

**Table 2:** Showing sensitivity, specificity at various cut off values and hour wise correlation of urine protein with 24 hours collection

**Hours**	**Total Urine protein** ** (mg) (Mean ± SD)**	**Cut-off value ** **proteinuria(mg)**	**No of Patients**	**Sensitivity**	**Specificity**	**r value** **(p value)**
2	112.9 ± 126.10	<25 >25	21 104	92.45%	68.42%	0.89 (<0.01)
4	227.5 ± 251.10	<50 >50	23 102	95.28%	94.74%	0.93 (<0.01)
8	408.1 ± 446.90	<100 >100	22 103	91.51%	84.21%	0.94 (<0.01)
12	652.4 ± 766.70	<150 >150	20 105	96.23%	84.21%	0.80 (<0.01)
24	994.4 ± 1159.30	<300 >300	19 106	------	------	-------

A sensitivity of 92.45%, 95.28%, 91.51%, and 96.23% and a specificity of 68.42%, 94.74%, 84.21% and 84.21% were obtained and correlation coefficient was 0.89, 0.93, 0.94 and 0.80 respectively and p value were <0.01 showing significant correlation (Table 2).

## Discussion

The present study was conducted on 125 patients in the Department of Obstetrics and Gynecology of Pt B D Sharma, Post Graduate Institute of Medical Sciences, Rohtak, India, to correlate the urinary protein levels in 2-, 4-, 8- and 12-hour urine samples with 24-hour urine sample in preeclampsia.

 In the present study, the mean maternal age (24.58 ± 3.90 years) was comparable with other studies by Rinehart et al. (25 ± 6.5 years) (8), Tara et al. (median age 25 years) (9), and Kieler et al. (10).

The gestational age in present study was comparable with study by Rinehart et al. who observed mean age of 29 ± 4.7 weeks (8). As per study by Adelberg et al., mean gestational age was 33.0 ± 2.8 weeks and 30.9 ± 2.1 weeks for mild and severe preeclampsia, respectively (11). Tara et al. conducted a study on 26 patients in which 92.3% were in third trimester and 7.7% were in second trimester of pregnancy (9). 

The average systolic and diastolic blood pressure was comparable with other studies (8, 9). Somnathan et al. (12), Tara et al (9) and Adelberg et al (11) observed proteinuria of 3+ or more in 16%, 12% and 18% of the subjects respectively.

Many studies have been carried out to study the correlation of level of proteinuria during different collection periods with that of 24 hour-urine protein. Rinehart et al. studied the correlation of two consecutive 12-hour urine samples with that of a 24-hour urine collection in 29 patients of preeclampsia and showed sensitivity of 96%, specificity of 100%, positive predictive value of 100%, negative predictive value of 80%, and a correlation coefficient of 0.89 (8). 

Kieler et al. compared urine albumin in spot and 12-hour urine samples with 24-hour urine collection in 30 women with preeclampsia. It was found that 12-hour collection correlated well with 24-hour collection, but the association of spot and 24-urine albumin was weak. So, they concluded that 24-hour urine collection can be substituted with 12-hour collection (10). Present study also showed strong correlation (p<0.01; Table 2) between 12- and 24-hour urine samples. 

Two other studies have shown a strong correlation of 8- and 12-hour urine protein with 24-hour urine protein (11, 13). In the present study, good correlation in 8-hour and 24-hour samples was observed (p<0.01; Table 2). Only one study by Wongkitisophon et al. (14) compared 4- and 24-hour proteinuria and found a significant correlation between the two (r= 0.95; p<0.001) (14). In the present study also a significant correlation was seen in 4-hour and 24-hour collection (Table-2). Two other authors have compared 2-hour protein creatinine ratio with 24-hour proteinuria and have concluded that 2-hour urine collection offers the same clinical information as 24-hour urine collection (12, 15). In the present study on comparing 2-hour urine protein with 24-hour urine protein, a statistically significant correlation (p <0.01) was found. 

It is observed that there is a significant correlation between 2-, 4-, 8-, 12- and 24-hour proteinuria. Thus, the evaluation regarding the severity can be conducted in a relatively short period. A shorter period to diagnose the severity of preeclampsia would have clinical benefits in deciding the timing of delivery and earlier use of antenatal glucocorticoids. Patient compliance will also improve because of shortened interval of collection.
